# A highly selective fluorescent probe for direct detection and isolation of mouse embryonic stem cells

**DOI:** 10.1016/j.bmcl.2015.06.037

**Published:** 2015-11-01

**Authors:** Yogeswari Chandran, Nam-Young Kang, Sung-Jin Park, Samira Husen Alamudi, Jun-Young Kim, Srikanta Sahu, Dongdong Su, Jungyeol Lee, Marc Vendrell, Young-Tae Chang

**Affiliations:** a Singapore Bioimaging Consortium, Agency for Science, Technology and Research, 11 Biopolis Way, #02-02 Helios, Singapore 138667, Singapore; b Department of Chemistry & MedChem Program, Life Sciences Institute, National University of Singapore, 3 Science Drive 3, Singapore 117543, Singapore; c MRC Centre for Inflammation Research, The University of Edinburgh, 47 Little France Crescent, Edinburgh EH16 4TJ, United Kingdom

**Keywords:** Mouse embryonic stem cells, Fluorescence imaging, Single cell PCR, Fluorescence-assisted cell sorting, Three germ layers

## Abstract

Stem cell research has gathered immense attention in the past decade due to the remarkable ability of stem cells for self-renewal and tissue-specific differentiation. Despite having numerous advancements in stem cell isolation and manipulation techniques, there is a need for highly reliable probes for the specific detection of live stem cells. Herein we developed a new fluorescence probe (**CDy9**) with high selectivity for mouse embryonic stem cells. **CDy9** allows the detection and isolation of intact stem cells with marginal impact on their function and capabilities.

## Graphical abstract

**Figure:**
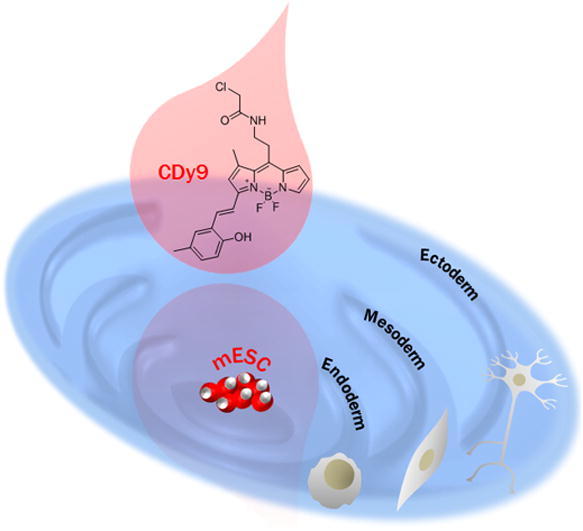

Stem cells are unique cell populations found in almost every multi-cellular organism with the capacity to give rise to many different types of cells in the body during early development and growth.^1–3^ Embryonic stem cells (ESC) are isolated from very young embryos (3.5 days-old) and found in the inner cell mass of blastocysts (i.e., embryos consisting of around 100 cells) of fertilized eggs.^3^ ESC are pluripotent as they are able to remain undifferentiated in vitro and then differentiate into all the different cell types of the three germ layers when given the correct cues.^4^

Despite the potential of stem cells for the treatment of complex diseases, there are still no effective ways to detect stem cells in vivo or ex vivo. This is mainly due to their heterogenetic nature and unpredictable pattern of proliferation and differentiation in ex vivo cultures.^2^ Current characterization methods for stem cells mainly rely on immunohistochemistry using protein markers and subsequent treatment with secondary antibodies.^5^ These methods typically require fixation of the cells with paraformaldehyde, which hampers the application of the cells in subsequent studies. Therefore, there is a need for new strategies that allow direct detection and monitoring of stem cells in a non-invasive manner.

Fluorescence imaging offers many advantages for non-invasive cell tracking. On top of being rapid and extremely sensitive, fluorescent probes are compatible with a broad range of instrumentation.^6^ In the last decade, considerable effort has been put into the development of highly sensitive fluorescent molecular imaging tools^7,8^ and genetically encoded fluorescent protein reporters.^9^ These reporters have proven their exceptional value in numerous biological studies, but entail some disadvantages, such as the potential interference with protein function and the need for genetic manipulation. An alternative to encoded protein reporters for labeling cells in vivo are small molecule fluorescent probes.^10,11^ Our group has pioneered the development of fluorescent probes using the Diversity Oriented Fluorescence Library Approach (DOFLA). DOFLA exploits the power of combinatorial chemistry to derivatize fluorescent scaffolds with several functional groups and generate libraries of structurally and spectrally diverse fluorescent molecules.^12^ To date, DOFLA has been an excellent source for the discovery of unique sensors and probes, especially for targets with limited molecular information.^13–15^ In the context of stem cell probe development, three fluorescent probes for mouse embryonic stem cells (mESC) have been previously identified by the DOFLA: compound of designation yellow 1 (**CDy1**), compound of designation green 4 (**CDg4**) and compound designation of blue 8 (**CDb8**).^16–18^ **CDy1** was the first mESC-staining probe to be reported from DOFLA, and it is a rosamine-based fluorophore that labels mESC as well as mouse induced pluripotent stem cells (iPSC). **CDy1** can stain mouse iPSC in early stages of development, allowing early detection and characterization of these cells.^19,20^ **CDb8** was reported as a mESC-staining probe with short emission wavelengths (*λ*_exc._/*λ*_em._:369 nm/487 nm) discovered upon the combinatorial derivatization of a xanthone fluorescent scaffold. Finally, **CDg4** is a chalcone derivative that labels mESC upon binding to the glycogen molecules present on the surface of mESC. Notably, despite the identification of these three fluorescent probes with preferential labeling of mESC over mouse embryonic fibroblasts (MEF), we observed that **CDy1**, **CDb8** and **CDg4** can also stain differentiated cells from various lineages (i.e., ectoderm, endoderm and mesoderm) and therefore cannot be used for direct and unequivocal identification and isolation of mESC.

Herein we report the identification of a fluorescent small molecule with high specificity for mESC and marginal staining in a whole range of cells derived from different lineages of the three germ layers. From our high-throughput cell imaging screening, we identified **CDy9** (compound of designation yellow 9, *λ*_exc._/*λ*_em._: 563 nm/578 nm) as a fluorescent small molecule with high selectivity for mESC over MEF (Fig. 1). **CDy9** was synthesized by derivatization of the 4,4-difluoro-4-bora-3*a*,4*a*-diaza-*s*-indacene (BODIPY) fluorescent scaffold using solid-phase synthesis (see Supporting information for synthetic and characterization data).^21^ In order to identify the chemical groups of **CDy9** that were responsible for its preferential labeling of mESC, we evaluated the fluorescence staining of mESC and MEF upon incubation with two chemical structures closely related to **CDy9** (Fig. S1). The examination of both the free-amine and the acetyl derivative confirmed **CDy9** as the most discriminatory compound of the family, indicating the relevance of the chloroacetyl group and highlighting the importance of acetylation of the *meso* aniline group of **CDy9** to achieve selectivity for mESC over MEF. **CDy9** is a highly versatile probe that enabled mESC detection and isolation using other fluorescence-based techniques, such as flow cytometry. As shown in Figure 1c, **CDy9** showed a significantly brighter signal in mESC when compared to MEF and to unstained mESC.

In view of the different staining properties of **CDy9** in mESC and MEF, we used our probe in co-cultures to evaluate its potential to simultaneously discriminate these two cell types. As shown in Figure 2a, **CDy9** brightly stained mESC in co-cultures with MEF. Furthermore, in order to validate our observation, we used fluorescence-assisted cell sorting (FACS) to isolate the two cell populations according to their side scattering (SSC) and **CDy9**-staining. We named the two populations as **CDy9**-dim and **CDy9**-bright cells, and analyzed their gene expression profiles by single cell PCR (Fig. 2d). Single cell PCR enables the characterization of rare events and small populations of cells with extremely high sensitivity.^22^ The gene expression profile of 48 individual brightly-stained cells and 48 individual dimly-stained cells was determined by a total of 33 primers from the TaqMan® Gene Expression Assay. Specifically, we selected 21 primers that are characteristic of pluripotent cells as well as 12 primers for differentiated cells. As shown in Figure 2c, the 21 selected genes (*red*) are upregulated in ESC and involved in stem cell initiation, maturation and stabilization. On the contrary, the other 12 genes (*blue*) are upregulated in differentiated cells (e.g., MEF) and widely considered as mesenchymal and differentiation markers.

We collected single cell PCR data for the two differently **CDy9**-stained populations, and plotted the resulting GAPDH-normalized values in a heatmap (Fig. 2d). From our heatmap, we concluded that **CDy9-**bright cells displayed a general trend in upregulation of pluripotent genes while **CDy9**-dim cells showed a tendency towards upregulating genes that are characteristic of differentiated cells. The heatmap plot also illustrates that ESC-representative genes (e.g., Oct4 (1), Esg1 (2)) showed uniformly high expression levels in **CDy9-**bright cells while the differentiation marker FN1 (33) was highly upregulated in almost all **CDy9**-dim cells. Further analysis by principle component analysis (PCA) corroborated the clear differences between **CDy9**-bright and **CDy9**-dim cells, and their correspondence to the gene expression profiles of mESC and MEF, respectively, (Fig. S2). Altogether, the results from both the heatmap and the PCA analysis validate the potential of **CDy9** as a fluorescent tool to specifically label mESC in cell mixtures.

Next we assessed whether **CDy9** could stain mouse iPSC obtained from MEF from transgenic mice expressing green fluorescent protein (GFP) under the Oct4 promoter control. GFP-expressing mouse iPSC were cultured and allowed to differentiate to generate complex mixtures of stem-like and differentiated cells.^23^ When we added **CDy9** to these complex mixtures, we observed that **CDy9** specifically stained non-differentiated mouse iPSC as observed from the co-localization with the GFP signal (Fig. S3a). Furthermore, we differentiated GFP-expressing mouse iPSC into mesoderm, ectoderm and endoderm lineages confirmed by immunohistochemistry with anti-SMA, anti-Nestin and anti-Sox17, respectively. **CDy9** and GFP signals were completely lost upon differentiation, which asserts the high selectivity of **CDy9** for mESC (Fig. S3b).

ESCs have the ability to prolong their self-renewal or differentiate depending on the signals from their microenvironment.^24^ In order to evaluate the ability of **CDy9** to selectively label mESC with marginal staining in differentiated cells, we screened our probe in several stem cells as well as in differentiated cells obtained from embryoid bodies. We included stem cell populations (e.g., mesenchymal stem cells and neural stem cells) in addition to mESC. Moreover, our panel included a wide range of cells from all three different germ layers since mESC can be readily differentiated into ectoderm, endoderm and mesoderm lineages (Fig. 3a).^25–27^

The endoderm is the innermost layer of the embryo. This layer consists of cells from the respiratory tract and the glands that are associated with the bladder, urethra and the gastrointestinal tract.

We selected three cell types from this lineage, namely pancreatic alpha, beta and acinar cells (Fig. 3c). The mesoderm is the middle layer that leads to the formation of muscles, bones, blood and connective tissues. Muscle C2C12 cells as well as T and B lymphocytes were chosen as representative cells from the mesoderm lineage (Fig. 3d). Finally, the ectoderm is the outermost layer of the three germ layers, which gives rise to cells from the central nervous system and epidermal tissues (e.g., skin). Our panel included NS5 differentiated astrocytes, primary neurons and mixed glial, which contains a mixture of microglia, astrocytes and oligodendrocytes (Fig. 3e). Notably, **CDy9** selectively stained only mESC with no detectable fluorescence emission in any of the other 11 cell types screened. We also compared the selectivity profile of **CDy9** to some of our previous mESC-staining fluorescent probes, namely **CDy1**, **CDg4** and **CDb8**. As shown in Figures S4–S6, while all these three compounds showed a clear and preferential staining in mESC, they also stained cells from some differentiated lineages (**CDy1**: endoderm and ectoderm; **CDg4**: endoderm, **CDb8**: endoderm).

Taken together, our results validate **CDy9** as a highly selective fluorescent probe for the detection and isolation of mESC using fluorescence microscopy and flow cytometry. In view of the potential of stem cells as invaluable tools for biological understanding, cell-based therapies and the development of novel drug discovery platforms,^28^ we believe that **CDy9** will accelerate those studies as a non-invasive biotechnological tool for the preparation, purification and characterization of mESC.

## Figures and Tables

**Figure 1:**
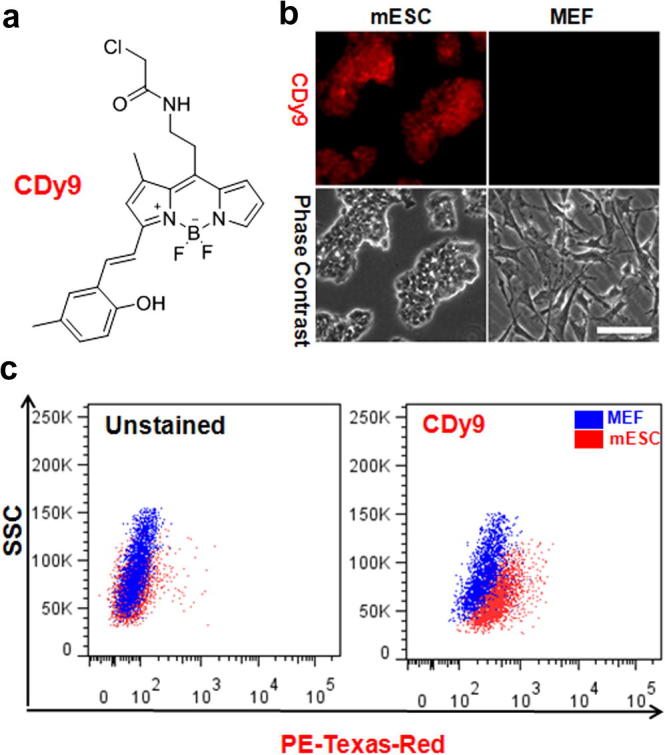
CDy9 is a novel and versatile mESC-specific fluorescent probe. (a) Chemical structure of **CDy9**. (b) **CDy9** selectively stains mESC over MEF. Both mESC and MEF were incubated with 1 μM **CDy9** and imaged under the fluorescence microscope after 1 h. Scale bar: 25 μm. (c) Flow cytometry analysis of mESC and MEF after incubation with **CDy9**. The fluorescence intensity of mESC upon treatment with **CDy9** is brighter than in MEF (right dot plot) and in unstained mESC (left dot plot).

**Figure 2:**
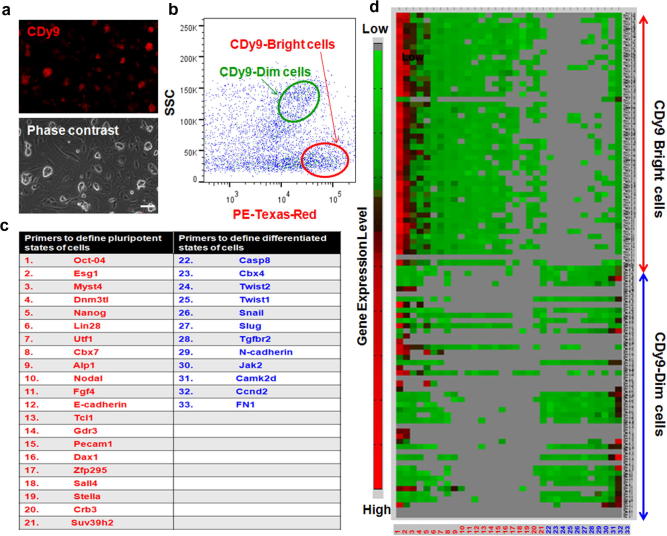
Isolation of CDy9-stained cells and analysis by single cell PCR. (a) **CDy9** selectively stains mESC in co-cultures with MEF. Co-cultures were incubated with 1 μM **CDy9** and imaged under the fluorescence microscope after 1 h. Scale bar: 100 μm. (b) FACS analysis of co-cultured mESC and MEF after **CDy9** staining. *X*-Axis refers to the **CDy9** fluorescence intensity and *Y*-axis refers to the side scattering (SSC), an indication of the size of the cells. FACS enabled the isolation of **CDy9**-bright and **CDy9**-dim populations. (c) Table of primers used to distinguish pluripotent (red) from differentiated (blue) cells. (d) Heatmap obtained from single cell PCR analysis of **CDy9**-bright and **CDy9**-dim populations. **CDy9**-bright cells display an up-regulation of pluripotent genes (mESC-like) and **CDy9**-dim cells show an up-regulation of differentiated genes (MEF-like).

**Figure 3:**
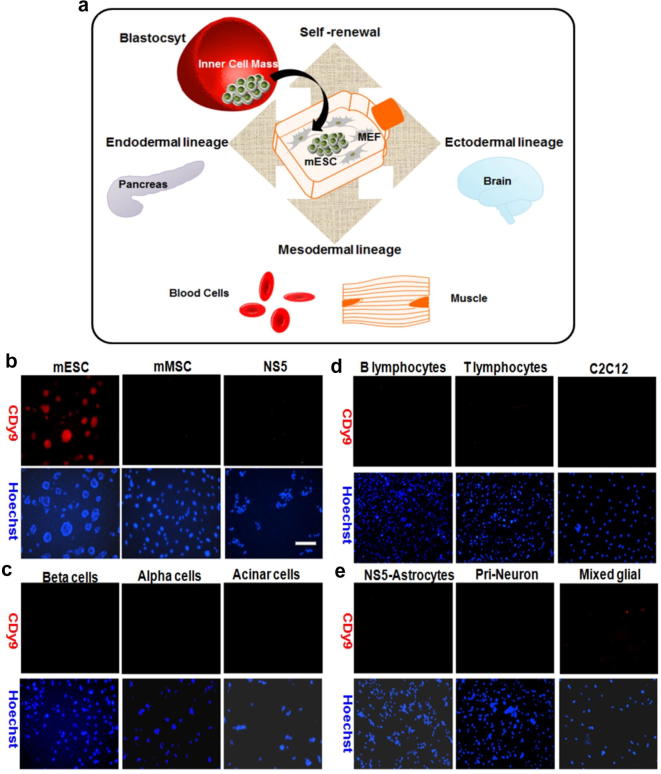
Diagram of pluripotent stem cell differentiation and cell panel test. (a) mESC are derived from the inner cell mass of a blastocyst and able to differentiate into all cells types of the three primary germ layers: mesoderm (e.g., muscle, blood), endoderm (e.g., pancreas), ectoderm (e.g., neurons). (b) **CDy9** staining in different stem cells: mESC, mouse mesenchymal stem cells (mMSC) and mouse neuronal stem cells (NS5). Scale bar: 200 μm. (c) **CDy9** staining in different pancreatic cells (endoderm). (d) **CDy9** staining in different muscle and blood cells (mesoderm). (e) **CDy9** staining in different neuronal cells (ectoderm). In all cases, cells were incubated with 1 μM **CDy9** and Hoechst 33342 (nuclear counterstain) and imaged under the fluorescence microscope after 1 h.
