# Supplementary material for: A highly selective fluorescent probe for direct detection and isolation of mouse embryonic stem cells
Source: Bioorg Med Chem Lett. 2015 Nov 1;25(21):4862–5. doi: 10.1016/j.bmcl.2015.06.037 (PMC4613884; doi:10.1016/j.bmcl.2015.06.037)
Supplement: Supplementary data — Supplementary material. [file mmc1.doc]

**Supplementary Material**

**A Highly Selective Fluorescent Probe for Direct Detection and Isolation of Mouse Embryonic Stem Cells**

Yogeswari Chandran, Nam-Young Kang, Sung-Jin Park, Samira Husen Alamudi, Jun-Young Kim, Srikanta Sahu, Dongdong Su, Jungyeol Lee, Marc Vendrell and Young-Tae Chang

**Supplementary Figure 1**. Fluorescence staining of **CDy9** derivatives in mESC and MEF.

**Supplementary Figure 2.** PCA analysis from single cell PCR data.

**Supplementary Figure 3.** **CDy9** can monitor the differentiation of mouse iPSC into different cell lineages.

**Supplementary Figure 4.** Representative cell panel test for **CDy1**.

**Supplementary Figure 5.** Representative cell panel test for **CDg4**.

**Supplementary Figure 6.** Representative cell panel test for **CDb8**.

**Materials and Methods**.


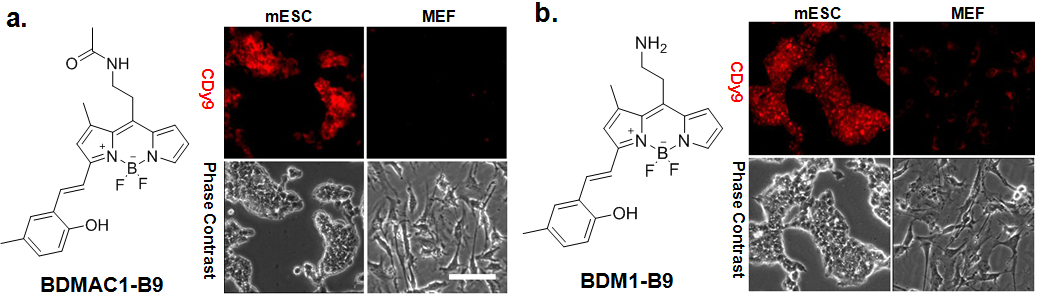
**Figure S1. Fluorescence staining of CDy9 derivatives in mESC and MEF**. a) Chemical structure of **BDMAC1-B9** (the corresponding acetyl-derivative of **CDy9**) and fluorescence staining in mESC and MEF. b) Chemical structure of **BDM1-B9** (the corresponding free-amine derivative of **CDy9**) and fluorescence staining in mESC and MEF. Both compounds were incubated in mESC and MEF at 1 μM for 1 h and imaged under the fluorescence microscope. Scale bar: 25 μm.


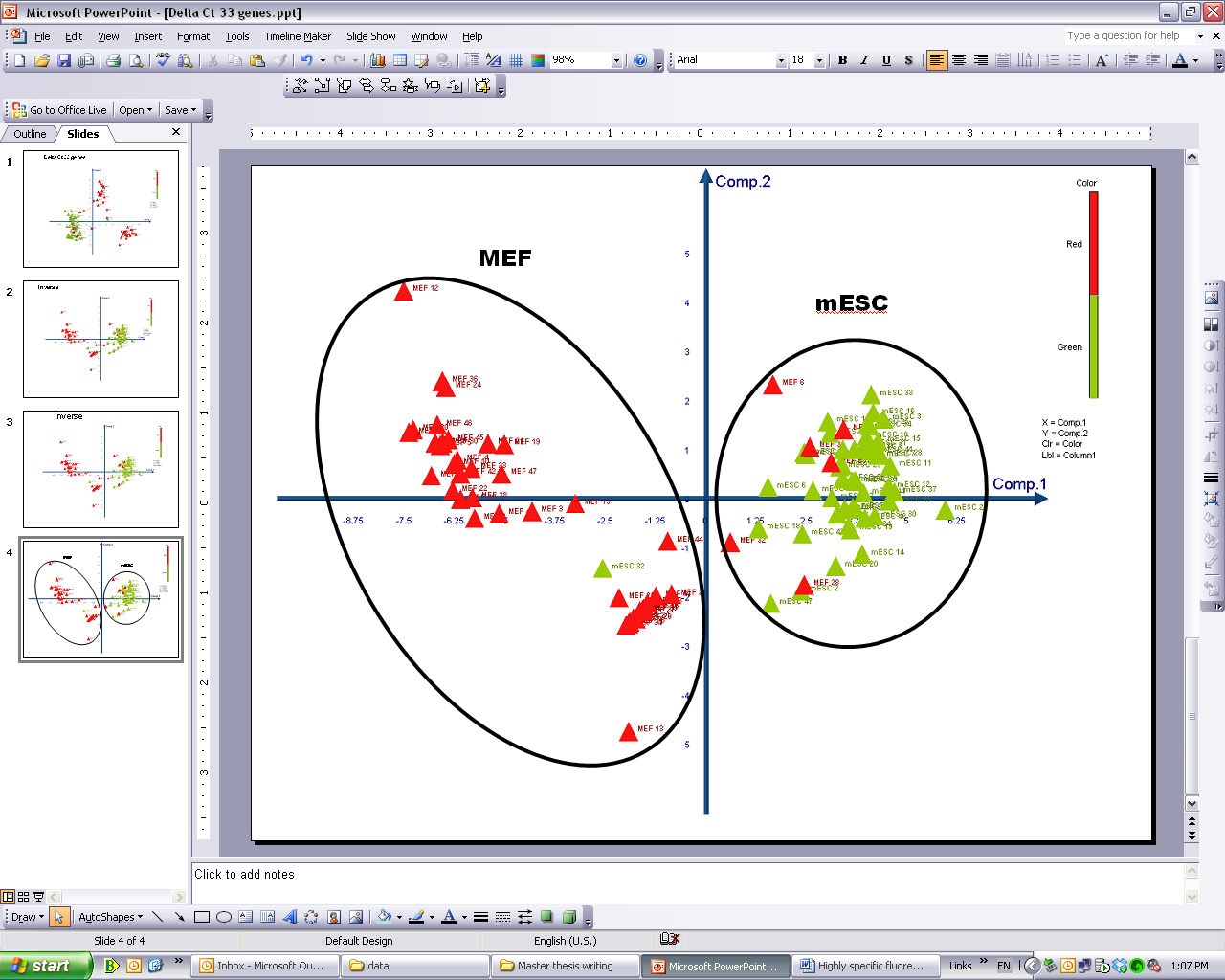
**Figure S2. PCA analysis from single cell PCR data**. The PCA plot shows that the **CDy9**-bright (*green triangles*) and **CDy9**-dim cells (*red triangles*) are clustered and clearly distinguishable by their **CDy9** staining.


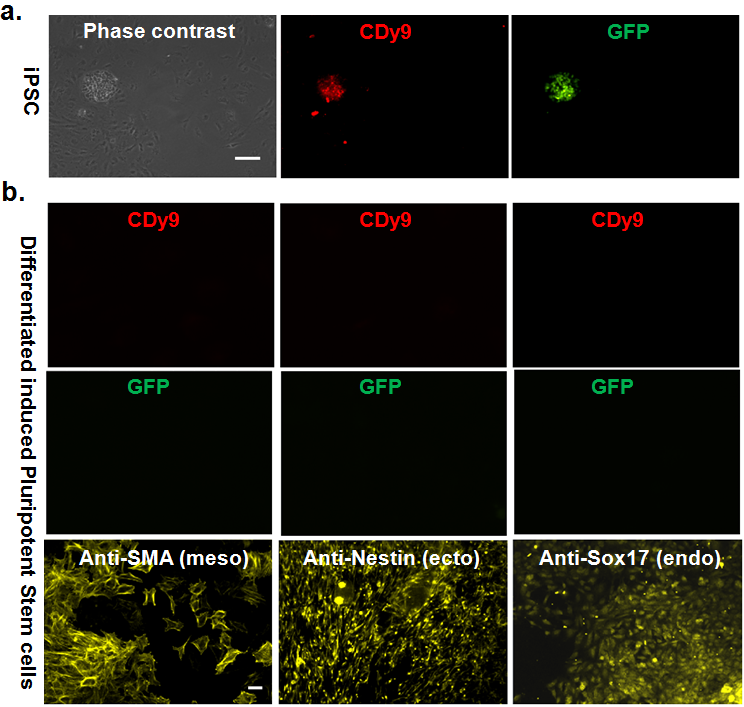
**Figure S3.** **CDy9 can monitor the differentiation of mouse iPSC into different cell lineages.** a) **CDy9** staining in mouse iPSC with GFP signal under the Oct4 promoter. 1 μM of **CDy9** was incubated with the cells for 1 h followed by image acquisition under a fluorescence microscope. Scale bar: 100 μm. B) Embryoid bodies from mouse iPSC were differentiated in media without LIF for 2 weeks and transferred to 12 well plates to allow the formation of monolayers. 1 μM of **CDy9** was incubated with the cells for 1 h followed by image acquisition under a fluorescence microscope. The formation of the three germ layers (Nestin-ectoderm, α-smooth muscle -mesoderm and Sox17-endoderm) was confirmed by immunohistochemistry.


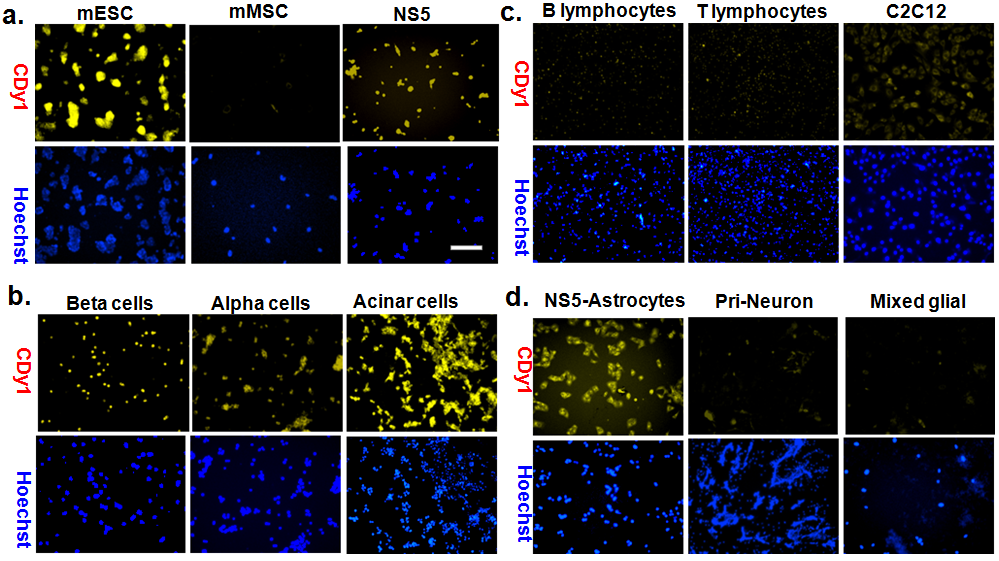
**Figure S4.** **Representative cell panel test for CDy1.** a) **CDy1** staining in different stem cells: mESC, mouse mesenchymal stem cells (mMSC) and mouse neuronal stem cells (NS5). Scale bar: 200 μm. b) **CDy1** staining in different pancreatic cells (endoderm). c) **CDy1** staining in different muscle and blood cells (mesoderm). d) **CDy1** staining in different neuronal cells (ectoderm). In all cases, cells were incubated Hoechst 33342 (nuclear counterstain) before being imaged under the fluorescence microscope.


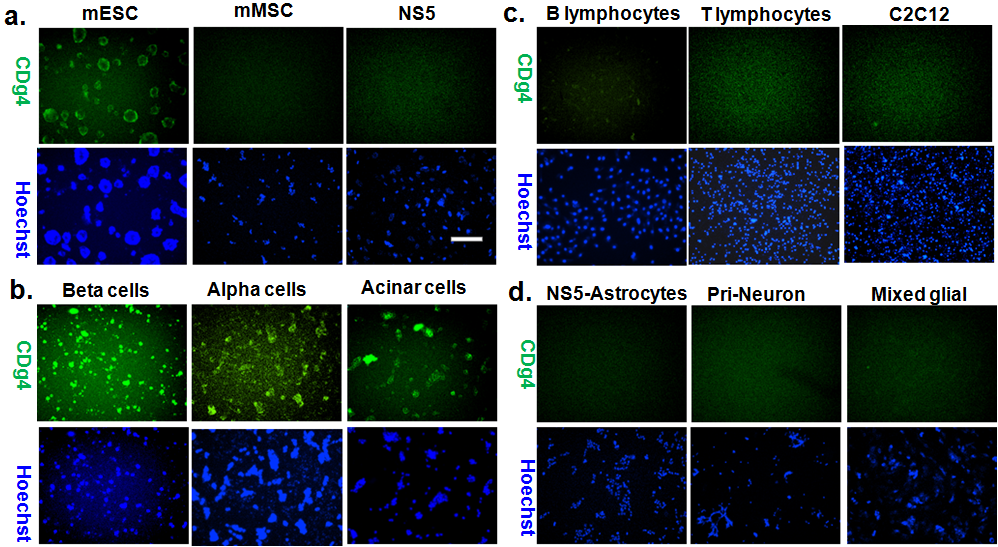
**Figure S5.** **Representative cell panel test for CDg4.** a) **CDg4** staining in different stem cells: mESC, mouse mesenchymal stem cells (mMSC) and mouse neuronal stem cells (NS5). Scale bar: 200 μm. b) **CDg4** staining in different pancreatic cells (endoderm). c) **CDg4** staining in different muscle and blood cells (mesoderm). d) **CDg4** staining in different neuronal cells (ectoderm). In all cases, cells were incubated Hoechst 33342 (nuclear counterstain) before being imaged under the fluorescence microscope.


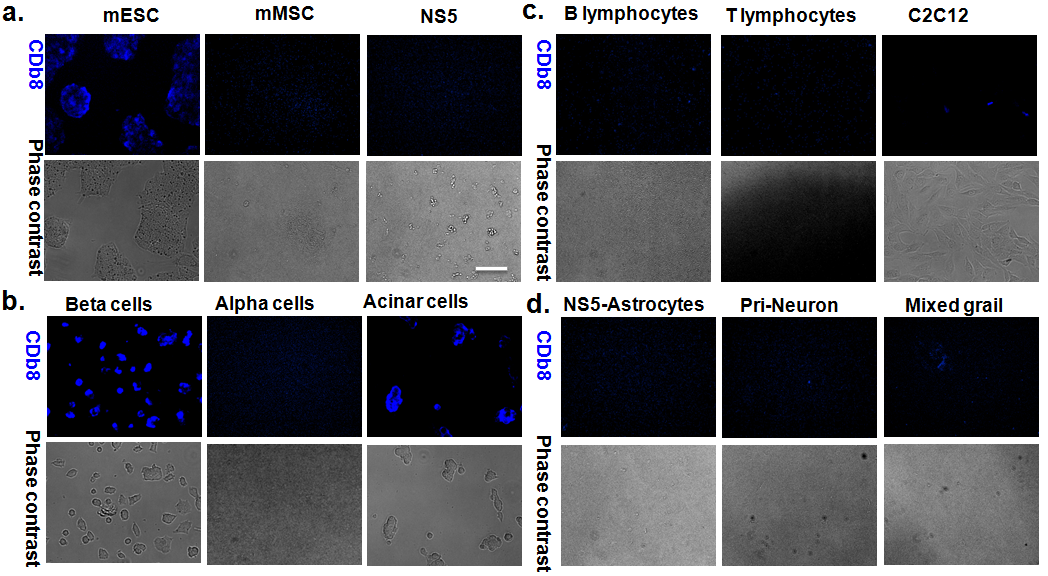
**Figure S6.** **Representative cell panel test for CDb8.** a) **CDb8** staining in different stem cells: mESC, mouse mesenchymal stem cells (mMSC) and mouse neuronal stem cells (NS5). Scale bar: 200 μm. b) **CDb8** staining in different pancreatic cells (endoderm). c) **CDb8** staining in different muscle and blood cells (mesoderm). d) **CDb8** staining in different neuronal cells (ectoderm). In all cases, cells were incubated Hoechst 33342 (nuclear counterstain) before being imaged under the fluorescence microscope.

**Materials and Methods**

mESC and MEF were provided by Sai Kiang Lim (Institute of Medical Biology, Singapore). Cells were passaged and stocks were made for subsequent use. All mice were provided by the Biological Resource Centre (BRC), A*STAR, with approval from the Institutional Animal Care and Use Committee (IACUC).

***Chemical synthesis and characterization of CDy9.***

Following a reported procedure (*Chem. Commun.* 2011, 47, 8424), the synthetic BODIPY intermediate (BDM) was dissolved in dry acetonitrile and mixed with 2-hydroxy-5-methylbenzaldehyde (4 eq.) and a 1:1 solution of pyrrolidine:AcOH (6 eq.). The mixture was refluxed at 85 ºC for 5 min, and the resulting amine-free conjugated BODIPY (**BDM1-B9**) was purified by silica gel chromatography using 98:2 dichloromethane:methanol. **BDM1-B9** was then dissolved in dichloromethane and a saturated solution of NaHCO_3_, followed by addition of chloroacetyl chloride (1.2 eq.) at 0 ºC. The reaction mixture was stirred at r.t. for 30 min to complete the formation of **CDy9**, which was purified by silica gel chromatography in 7:3 hexane:ethyl acetate.

Characterization data for **CDy9**:

^1^H-NMR (DMSO-d_6_, 300 MHz): δ 1.5 (m, 2H), 2.0 (m, 2H), 2.3 (s, 3H), 2.6 (s, 3H), 4.1 (s, 2H), 6.5 (m, 1H), 6.8 (m, 1H), 7.05 (m, 1H), 7.2-7.3 (m, 3H), 7.5-7.8 (m, 3H), 8.7 (m, 1H), 10.1 (s, 1H).


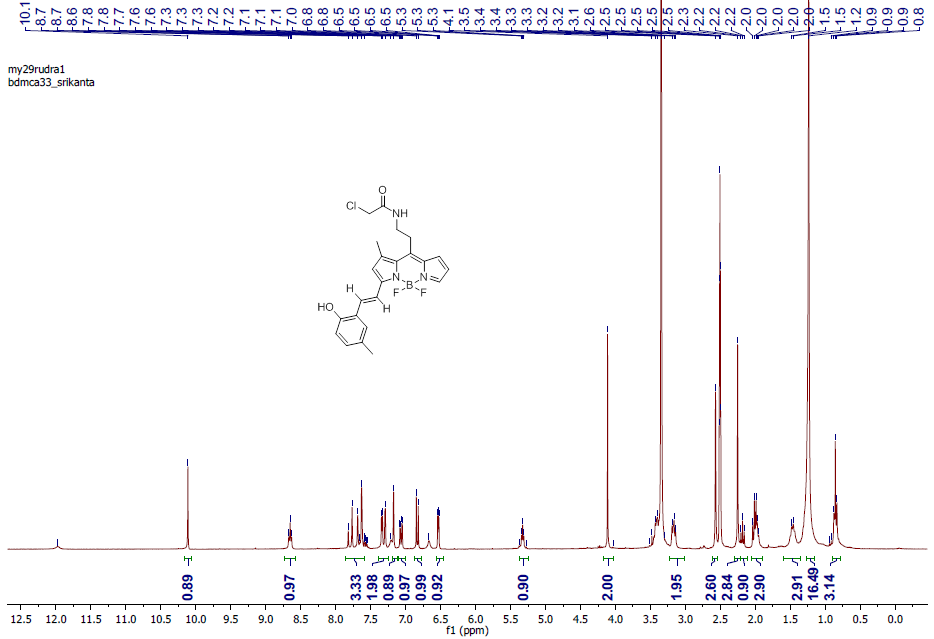


^13^C-NMR (CDCl_3_, 125 MHz): δ 14.3, 16.2, 20.4, 22.5, 42.9, 116.1, 116.7, 117.8, 120.4, 122.5, 123.5, 128.5, 130.0, 132.5, 134.2, 135.2, 136.9, 140.7, 145.7, 155.0, 158.2, 166.6, 174.6.


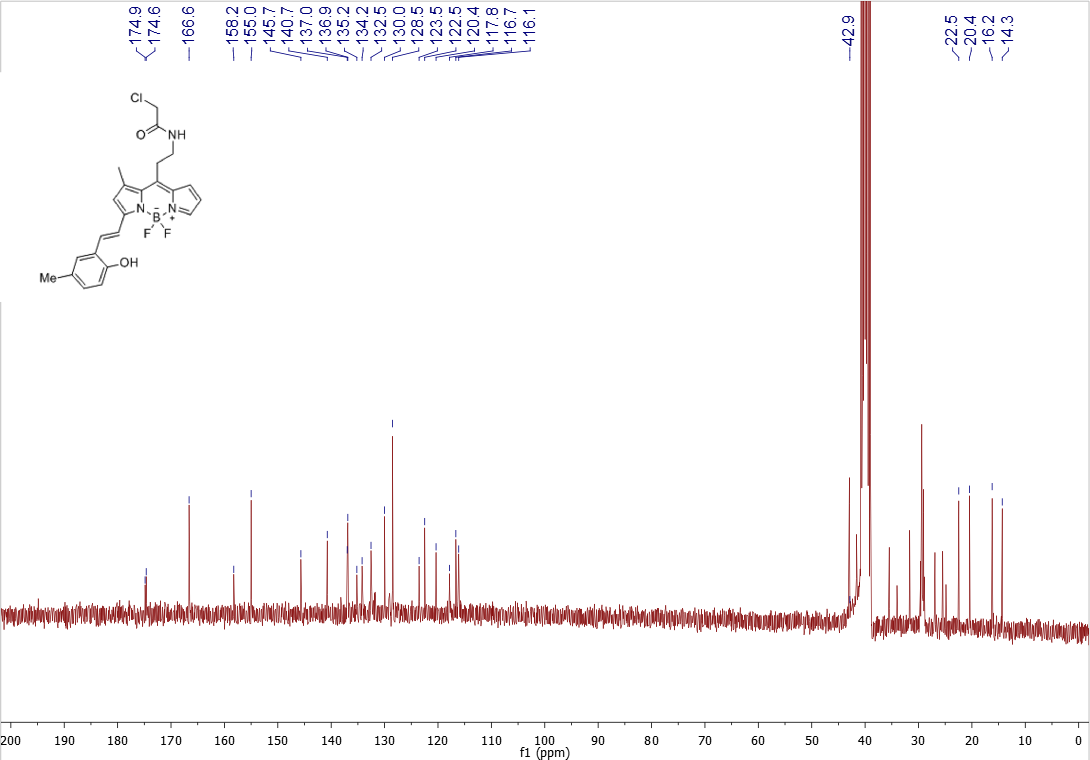


HRMS: m/z calculated for C_23_H_23_BClF_2_N_3_NaO_2_: 480.1436; found: 480.1439.

***High-throughput cell imaging screening.***

MEF were used as the negative control for the identification of mESC-selective probes, because most stem cells require a layer of feeder cells to provide the structural support for their proliferation and undifferentiated growth. Therefore, an ideal mESC-probe will stain mESC but not MEF. Specialized media were prepared for the two cell types. Dulbecco’s Modified Eagle’s Medium (DMEM) with 4.5g/L glucose (Gibco), 20% Fetal Bovine Serum (FBS) (Gibco), 1 % Non Essential Amino Acid (NEAA) (Gibco), 0.1% beta-mercaptoethanol (Gibco) and 1% Penicilin Streptomycin Glutamate (PSG) (Gibco) were added together to prepare the media for MEF cells. The same media was used for mESC with the addition of Leukemia Inhibitory factor (LIF) to prevent differentiation of the cells. 384 well-plates (Greiner) were used for the high-throughput cell imaging screens. 0.1% Gelatin in sterile water (Merck- ES-006-B) was used to coat the plates before seeding of mESC to enable adherence of the cells to the wells. Plates were incubated with 50 μL of 0.1% Gelatin for 30 min before removal and seeding of the cells into the appropriate well. The cells were plated in the following order: MEF, mESC and a co-culture of mESC and MEF. Based on the size of the cells, 1×10^5^ cells/mL mESC and 2.5×10^4^ cells/mL MEF were seeded per well. Cell viability was checked by Trypan Blue staining and counted using a haemocytometer before seeding into the wells. Cells were incubated for 1 day to settle before addition of the fluorescent compounds for imaging. 1 μM of compounds previously dissolved in dimethyl sulfoxide (DMSO) were added to the wells with 1 h incubation at 37 °C before image acquisition using a high-content automated imaging microscope (ImageXpress, Molecular Devices). 1 μg/mL Hoechst 33342 was added per well for nuclei counterstaining. After image acquisition, images were analyzed using the MetaXpress High Content Image Acquisition & Analysis Software. The selected pool of hit compounds was further narrowed down via subsequent secondary and tertiary screening.

***Flow cytometry analysis.***

1×10^5^ cells/mL mESC and 2.5×10^4^ cells/mL MEF cells were cultured in 6 well-plates until they reached confluency. 1 μM of compounds were added to the wells with 1 h incubation at 37 °C. After incubation, cells were washed with Phosphate Buffered Saline (PBS) 3 times. Cells were then treated with trypsin (Invitrogen) for 5 min before neutralizing with the appropriate culture media and centrifuging at 1,500 rpm for 3 min in 5 mL tubes. PBS washing was carried out 3 more times with centrifugation. Finally, 500 μL of PBS was added per tube. Unstained MEF and mESC were used as controls. A BD LSR II Bioanalyzer (BD Biosciences) was used to analyze the samples. 10,000 cells were analyzed per tube and data was processed using Flowjo (Tree Star, Inc).

*C****o-cultures of MEF and mESC.***

Co-cultures of MEF (2.5×10^4^/mL) and mESC (1×10^5^/mL) were prepared in gelatin-coated 6 well plates. 1 μM **CDy9** was added with 1 hour incubation at 37 °C. After 1 h, cells were washed 3 times with PBS and treated with trypsin for 5 min at 37 °C. Cells were neutralized with culture media before centrifugation at 1,500 rpm for 3 minutes. The cells were washed 3 more times with PBS before adding a final volume of 500 μL of PBS in 5 mL FACS tubes. Stained and unstained individually grown MEF and mESC were used as controls. The FACS Aria IIu SORP Cell sorter was used for cell sorting. **CDy9**-bright and **CDy9**-dim populations were gated and a total of 100,000 cells were collected from dim and bright cells.

***Single cell PCR***

Single cell PCR was carried out to determine the expression of genes in different population of cells sorted from co-cultures of mESC and MEF stained with **CDy9**. As such, a co culture of MEF (2.5×10^4^/mL) and mESC (1×10^5^/mL) were mixed and 1 μM **CDy9** was added with 1 hour incubation at 37 °C. After incubation, the cells were prepared for FACS sorting. 96 well-plates (Sorenson-28440) with 10 μL of master mix per well were employed for single cell sorting.

|  | **For 1 sample** |
| --- | --- |
| CellsDirect 2×Reaction Mix | 5 μL |
| 0.2×Assay pool (contains primers and ultrapure water) | 2.5 μL |
| RT/ Taq enzyme | 0.5 μL |
| TE buffer/ Ultrapure water | 2 μL |
| Total | 10 μL |

Bright and dim populations were gated and 1 cell/well was sorted into 96 well-plates. Plates were then stored at -80 °C for 1 h and thawed to promote cell lysis. Next, reverse transcription and cDNA amplification was carried out using the following settings:

- 1. 50deg, 20 min (reverse transcribe RNA to cDNA)
  2. 95deg, 2 min (inactivate RT enzyme and start Taq)
  3. 95deg, 15 sec
  4. 60deg, 4 min
  5. Repeat c and d for 17× (pre-amplify cDNA by denaturing for 18 cycles (13 cycles for single spheres) at 95deg for 15 sec each, and annealing at 60deg for 4 min).
  6. 8deg

40 μL of nuclease-free water was added per well and the amplified cDNAs were stored at -80 °C. On the day of the experiment, 10 μL of 2×DA assay loading reagent (Fluidigm, PN 85000736) was added into each of the 48 PCR tubes, containing 10 μL of the respective Taqman probe. The pre-sample mix was then prepared containing 182 μL of Taqman® Universal PCR Master Mix (Applied Biosystems, PN 4304437) and 18.2 μL DA sample loading reagent (Fluidigm, PN 85000735). Lastly, 3.8 μL of the pre-sample mix was added to the 3.2 μL of respective diluted cDNA before priming the 48 sample*48 assay integrated fluidic circuit (IFC) with tuberculin. Next 5 μL of the real–time assay mix and 5 μL of the respective final sample mix were loaded into the chambers and the sample was loaded into the BioMark™ HD System.

***Differentiation of miPSC to ectoderm, endoderm and mesoderm and staining using CDy9***

mouse iPSC were prepared as reported by Yamanaka et al. (Cell, 2006). Oct4-tagged MEF cells were used for mouse iPSC production so that the presence of iPSC would be confirmed by the GFP signal. 1 μM **CDy9** was added with 1 hour incubation at 37 °C followed by fluorescence microscopy imaging. Embryoid bodies were prepared from mouse iPSCs by seeding them on low adherent plates with MEF media for 4 days. 5-10 colonies were transferred into 6 well-plates and cells were allowed to grow until they attached and differentiated. 1 μM **CDy9** was added again to confirm fluorescence staining. Immunocytochemistry was performed to identify mesoderm, ectoderm and endoderm lineages.

Mesoderm: Primary antibody: Mouse mAb [0.N.5] to α-smooth muscle actin (AB18147 – Abcam)

Secondary antibody: Cy5 goat anti-mouse IgG (H+L) (A10524 – Invitrogen)

Ectoderm: Primary antibody: Anti-Nestin, clone rat-401 (MAB353 – Millipore)

Secondary antibody: Cy5 goat anti-mouse IgG (H+L) (A10524 – Invitrogen)

Endoderm: Primary antibody: Anti-hSOX17 (AF1924- R&D systems)

Secondary antibody: Alexa Fluor 647 donkey anti-goat IgG (H+L) (A21447 – Invitrogen)

***Cell Panel Screening***

A total of 12 cell types including mESC were collected for the screen. Four cell types, namely α-cells, β-cells, acinar cells and mouse mescenchymal stem cells were chosen as representatives for the endoderm lineage. Four cell types (NS5, NS5 derived astrocytes (NS5-D), primary neurons and mixed glial) were chosen for the ectoderm lineage. C2C12, B cells and T cells were chosen as representatives of the mesoderm lineage. All cells were seeded in 384 well-plates and compounds were added to the cells at 1 μM. 1μg/mL Hoechst 33342 was added 15 min prior to image acquisition for nuclei staining. Cell images were taken using the ImageXpress and analyzed using the MetaXpress High-Content Image Acquisition & Analysis Software.
